# A method for the permeabilization of live *Drosophila melanogaster* larvae to small molecules and cryoprotectants

**DOI:** 10.1080/19336934.2020.1724051

**Published:** 2020-02-09

**Authors:** Alex Murray, Daniel Palmer, Daimark Bennett, Venkata Dwarampudi, João Pedro de Magalhães

**Affiliations:** a Institute of Ageing & Chronic Disease, University of Liverpool, Liverpool, UK; b Institute of Integrative Biology, University of Liverpool, Liverpool, UK; c Division of Biomedical and Life Sciences, Lancaster University, Lancaster, UK

**Keywords:** *Drosophila*, larvae, permeabilization, imaging, cryobiology, cryopreservation, method

## Abstract

The larvae of *Drosophila melanogaster* is a model organism widely used to study the muscular and nervous systems. *Drosophila* larvae are surrounded by a waxy cuticle that prevents permeation by most substances. Here we develop a method to remove this layer, rendering the larvae permeable to small molecules without causing death, allowing the larvae to develop to adulthood and reproduce. Permeability was assessed using fluorescein diacetate dye uptake, and mortality upon exposure to toxic levels of ethylene glycol (EG) and Dimethyl sulfoxide (DMSO). Potential uses for this method include drug delivery, toxicity assays, cryopreservation, staining, and fixation.

## Introduction

The common fruit fly *Drosophila melanogaster* is a widely used model organism, favored for its low cost, short generation time and extensively studied genome. Its larvae have multiple uses, including the study of inflammation [1], cancer biology [2,3], and drug discovery [4]. Advantages of using *Drosophila* larvae include the ability to visualize the larvae’s developing muscular and nervous systems using florescent microscopy, which is possible because the larvae are translucent [5].

In drug discovery, a common method of investigation is to expose a living system to the drug and monitor its effects on a protein or molecular system. In cell and tissue culture this is simple; cells can be exposed to drugs by bathing the cells directly in medium containing the compound of interest. However, *Drosophila* larvae are surrounded by a waxy epicuticular layer [6]. This layer is impermeable to most substances, including water and other small polar molecules. A method has been developed to permeabilize *Drosophila* embryos [7] using a 1:1 solution of heptane and limonene. This treatment allowed the delivery of nocodazole into the internal tissues of the embryos. Upon attempting to use this method on larvae, we found that it invariably led to rapid larval death. Here we have adapted Schulman, Folker and Baylie’s protocol, finding an optimal solution and treatment time for use in live larvae.

## Results

### Optimized permeabilization protocol

Our first attempts to permeabilize *Drosophila* larvae used a permeabilization solution consisting of 50% heptane 50% D-limonene with an exposure time of 100 seconds. This solution was developed by Schulman et al. [7] to permeabilise *Drosophila* embryos. These attempts were successful in permeabilizing larvae to fluorescein diacetate (FDA), a fluorescent tracker dye, however, in each attempt all larvae were killed in the process. Subsequently we developed a non-lethal protocol. It was found that third instar larvae exposed to 10% heptane 90% D-limonene for exactly 45 seconds became permeable to FDA without causing death.

The full protocol is as follows:

1. Obtain third instar larvae using a handling needle or tweezers. Place them onto a glass dissecting dish (limonene can damage plastic containers).
2. Cover the larvae in about 300 µl of permeabilization solution (10% heptane, 90% limonene) for exactly 45 seconds. Longer treatment times result in a high proportion of the larvae being killed. It is important to remove the solution as fast as possible after treatment.
3. Aspirate and discard permeabilization solution, then quickly blot dry the larvae with tissue paper to remove excess solution.
4. Immediately cover the larvae with the substance they are to take up. Exposure time must be optimized for specific substances.

### Larvae can be permeabilized to fluorescein diacetate

**Figure 1.**
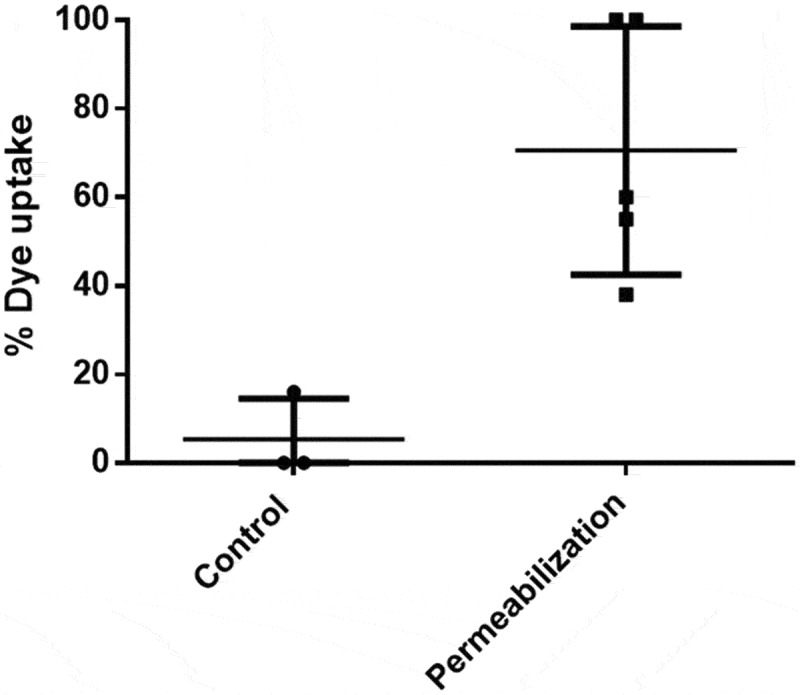
Third instar larvae were treated with 300µl permeabilization solution (10% heptane, 90% limonene) for 45 seconds before soaking in fluorescein diacetate for 20 minutes. 70.6% of individual treated larvae were permeabilized to FDA compared with 5.3% of control larvae

**Figure 2.**
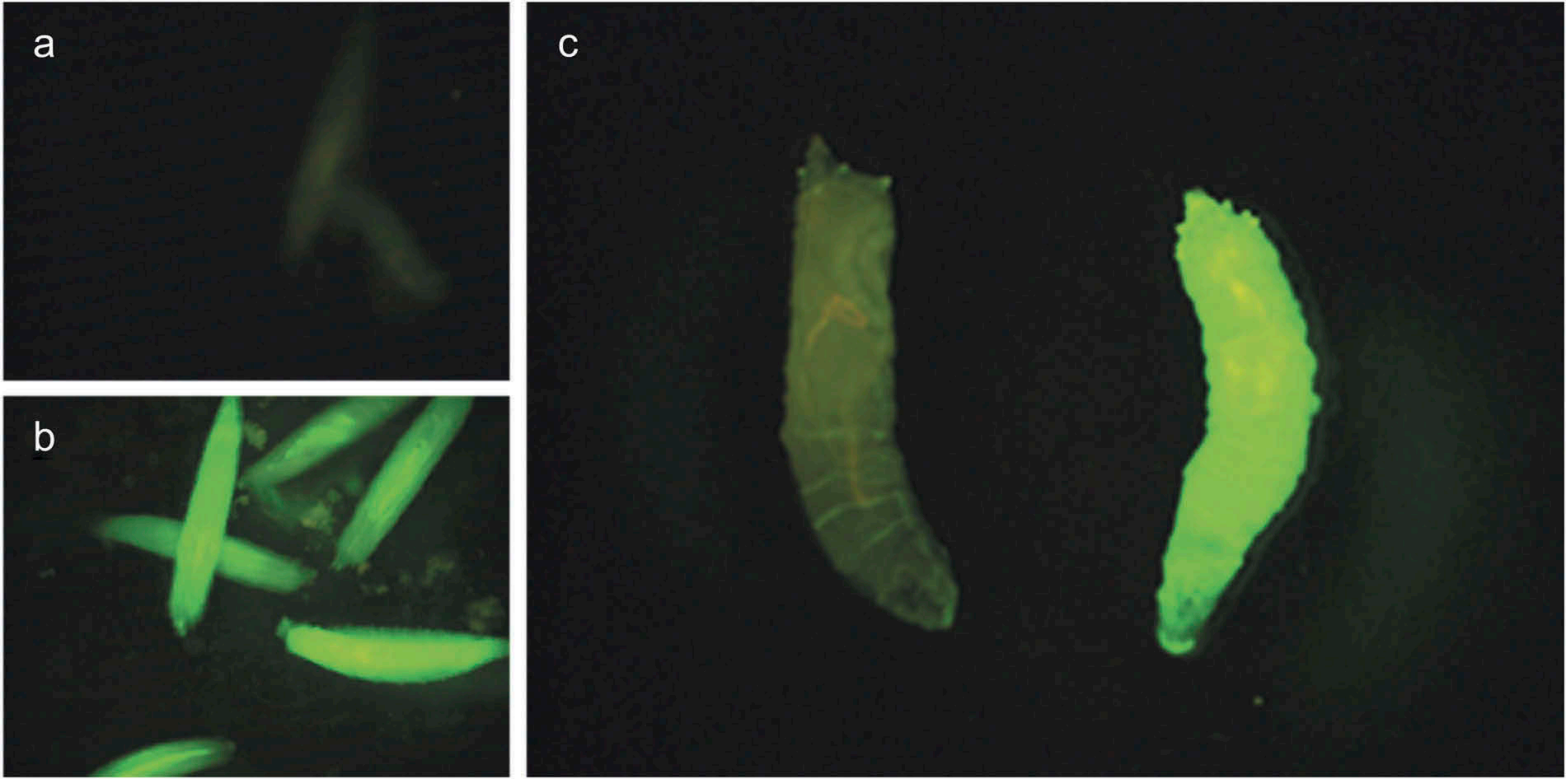
Fluorescein diacetate uptake was visualized using florescence microscopy at 488 nm. (a) Live non-permeabilized larvae. (b) Live permeabilized larvae. (c) Culled non-permeabilized (left) and permeabilized (right) larvae side by side

Fluorescent microscopy was used to measure fluorescein diacetate uptake after 20 minutes. Larvae were considered to have taken up the dye if it could be visibly seen within the larval tissue. It was found that 70.6% of treated larvae (n = 44 across 5 batch repeats) were permeable to FDA compared with 5.3% of non-treated control larvae (n = 26 across 3 batch repeats) (p = 0.009) (Figures 1 and 2).

### Permeabilized larvae are able to survive to adulthood and successfully reproduce

**Figure 3.**
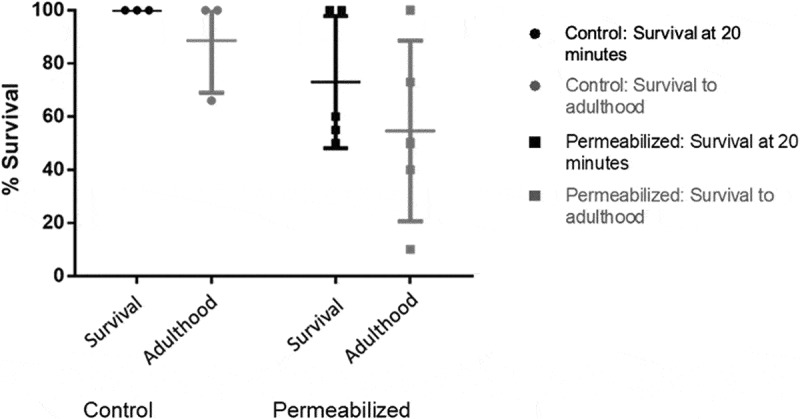
Larvae were monitored for short term survival, as measured by observing the larvae wriggling, and development to adulthood (labeled as ‘survival’ and ‘adulthood’ on the graph respectively). 73.0% of permeabilized larvae survived for at least 20 minutes and 54.6% of permeabilized larvae survived to adulthood after the permeabilization process

Permeabilized larvae were monitored for immediate survival (defined by wriggling after 20 minutes) and development to adulthood. After development to adulthood, the flies were counted and pooled into a single vial where they were allowed to breed. It was found that 73.0% of permeabilized larvae survived 20 minutes after the permeabilization process compared with 100% of control larvae, and 54.6% of permeabilized larvae survived to adulthood compared with 88.7% of control larvae. These differences were not statistically significant (p = 0.498 and p = 0.311 respectively) (Figure 3). Surviving flies were observed to successfully breed with each other and produce a new generation of adult flies.

### Larvae were permeabilized to ethylene glycol and DMSO

**Figure 4.**
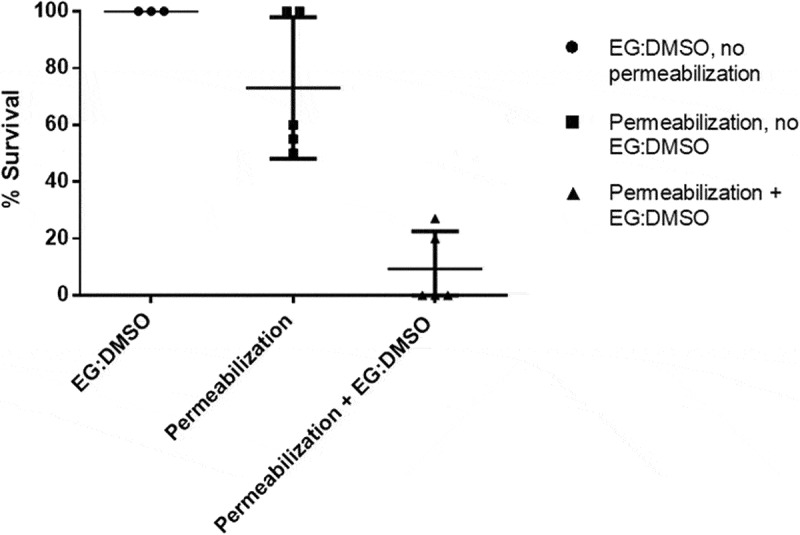
Larvae were treated using permeabilization solution (10% heptane, 90% limonene) and then exposed to 50% CPA solution (25% EG, 25% DMSO in DPBS). 9.4% of larvae survived when treated with permeabilization solution and soaked in CPA solution, compared with 100% of those soaked in CPA solution without treatment, and 73% of those treated with permeabilization solution without soaking in CPA solution

We then tested the ability of our protocol to permeabilize larvae to cryoprotective agents (CPAs). Larvae were treated with permeabilization solution and then soaked in CPA solution (25% EG, 25% DMSO in DPBS (v/v)) for 20 minutes. This concentration of CPAs is toxic at room temperature and without a proper loading ramp, thus larval mortality here is a measure of the extent to which they are permeable to the CPAs. Non-treated larvae were also soaked in CPA solution for 20 minutes and used as a negative control. Survival data from treated larvae which were soaked in the nontoxic FDA solution was used as an internal control (n = 44 across 5 batch repeats, as above). It was found that just 9.4% of larvae (n = 52 across 5 batch repeats) survived when treated with permeabilization solution and then soaked in CPA solution, compared with 100% of those soaked in CPA solution without treatment (n = 17 across 3 batch repeats) (p = 0.0001), and 73% of those treated with permeabilization solution without soaking in CPA solution (p = 0.0006) (Figure 4).

## Discussion

We have developed a method for permeabilizing live *Drosophila* larvae. While methods for permeabilizing dead larvae and live embryos already exist [7,8], this is the first protocol which allows for live larvae to be permeabilized without killing them, allowing them to develop to adulthood and reproduce. It should be noted that while the decrease in survival (Figure 3) was not statistically significant, this is likely due to the relatively low sample size (up to 5 experiments) and high variability of the survival rate. Thus, it is likely that permeabilization does negatively affect larval survival and development to adulthood. While it is easy to acquire larvae in sufficient numbers to ensure that many will survive for further experimentation, it is advisable to use a permeabilized control group if toxicity is a relevant experimental outcome. A second limitation of our method is that survival rate is heavily dependent on the exact time that the larvae are exposed to the permeabilization solution; anecdotally, a few seconds of over-exposure to permeabilization solution can result in all of the larvae being killed. The relationship between dye uptake and dye application time was not explored, but dye uptake likely follows standard diffusion mechanics, whereby internal dye concentration increases with time until an equilibrium is reached. A longer incubation time with heptane/limonene is almost always lethal, but the dead larvae invariably take up a large amount of dye. The same (high lethality, high dye uptake) is observed when the proportion of heptane is increased to 50%. Thus, dye uptake is (likely) proportional to treatment time, dye incubation time, and the proportion of heptane in the solution. Further optimization attempts may benefit from decreasing the concentration of heptane below 10% – which may decrease the time-sensitivity of the treatment, making the optimal treatment time longer but less critical.

We have demonstrated that the treated larvae are permeable to fluorescein diacetate, and a CPA solution containing ethylene glycol and DMSO. It is likely that our method renders larvae permeable to many other molecules, meaning that there are a variety of potential uses for this protocol: Firstly, drugs and other substances of interest could be delivered into the larval tissues, which is useful for introducing substances that cannot be absorbed through the larval digestive tract. This has applications in toxicity testing and drug discovery. Secondly, it may be possible to stain permeabilized larvae using tissue specific dyes, allowing for the live imaging of certain internal structures. Thirdly, as the larvae are permeabilized to ethylene glycol and/or DMSO, a cryopreservation protocol for *Drosophila* larvae may be developed by introducing CPAs into the larval tissues [9]. Finally, we have found that permeabilization allows larvae to be fixed using paraformaldehyde to preserve them for subsequent staining and imaging. While our method seems to remove the outer wax layer of the larvae, any molecules to be taken up must still be able to penetrate cellular and basement membranes. This size/polarity limitation might be overcome by combining our method with other mechanisms of delivery which are more commonly deployed in cell biology; such as liposomes, nanoparticles, polymers, electroporation and carrier peptides [10].

## Methods

### Drosophila handling

Flies (wild caught *Drosophila melanogaster*, Lancaster, UK) were cultured on sugar/maze agar medium (8.5% sugar, 6% maze mele, 10% agar, 2% autolyzed yeast, 3% propionic acid, 2.5% nipagin), contained within 50ml plastic vials at 25ºC with a 12-hour day/night cycle.

### Development of the permeabilization solution and protocol

Larvae of various stages were collected and placed into a glass dissecting plate. Larvae were then exposed to 10–50% heptane diluted in D-limonene (both Sigma) for time periods between 100 and 45 seconds. After treatment, larvae were soaked in 62.5 µg/ml fluorescein diacetate (FDA) in DPBS. Fluorescein diacetate uptake was imaged using florescent microscopy at 488 nm with a Leica MZ10F microscope and Leica Las X software. Surviving larvae were transferred to fresh media and monitored for development to adulthood.

### Cryoprotectant toxicity assay

Larvae were permeabilized using the optimized method above. They were then exposed to CPA solution (25% EG, 25% DMSO in DPBS) (all Sigma) for 20 minutes. Larvae were observed to determine survival, defined as continued wriggling.

### Statistics

Prism 6 (GraphPad) was used to perform statistical analysis and produce figures. A two tailed T-test was used to analyze FDA dye uptake (Figure 1). ANOVA was used for survival after permeabilization (Figure 3) and the application of CPAs (Figure 4) using Tukey’s multiple comparisons test and Geisser-Greenhouse correction. p-values below 0.05 were considered statistically significant.
